# Metabolome Profiling in Aging Studies

**DOI:** 10.3390/biology11111570

**Published:** 2022-10-26

**Authors:** Elena E. Balashova, Dmitry L. Maslov, Oxana P. Trifonova, Petr G. Lokhov, Alexander I. Archakov

**Affiliations:** Institute of Biomedical Chemistry, Pogodinskaya St. 10, 119121 Moscow, Russia

**Keywords:** metabolome profiling, aging, animal models, human

## Abstract

**Simple Summary:**

Low-molecular-weight substances are participants in all biochemical processes occurring in the body. Therefore, by measuring them we can obtain new knowledge about aging mechanisms. At the same time, various animals, which are distinguished by different life expectancies, are excellent objects for such studies, and modern science, known as metabolomics, offers efficient methods to measure them, taking into account their huge diversity. This review describes the aging data accumulated today, obtained by such methods in various animal models and humans.

**Abstract:**

Organism aging is closely related to systemic metabolic changes. However, due to the multilevel and network nature of metabolic pathways, it is difficult to understand these connections. Today, scientists are trying to solve this problem using one of the main approaches of metabolomics—untargeted metabolome profiling. The purpose of this publication is to review metabolomic studies based on such profiling, both in animal models and in humans. This review describes metabolites that vary significantly across age groups and include carbohydrates, amino acids, carnitines, biogenic amines, and lipids. Metabolic pathways associated with the aging process are also shown, including those associated with amino acid, lipid, and energy metabolism. The presented data reveal the mechanisms of aging and can be used as a basis for monitoring biological age and predicting age-related diseases in the early stages of their development.

## 1. Introduction

In recent decades, the lifespan (LS) of people in the world has been steadily increasing against the backdrop of a declining birth rate [1]. Population aging has become a global phenomenon and, perhaps, among the most significant social transformations of the 21st century, resulting in problems for the economy, as well as social problems, in particular for health care, since aging is often accompanied by disability, cardiovascular diseases, chronic respiratory diseases, Alzheimer’s disease, arthritis and diabetes [2,3]. Thus, it became necessary to study the molecular mechanisms of aging to reduce or eliminate the symptoms associated with it [4].

During aging, many transformations occur in the body at all levels of its organization, from cell organelles to organ systems, which lead to a wide range of functional and structural changes. However, this process is far from being fully studied. Over the past 30 years, gerontological studies have led to impressive advances in understanding the genetic control of aging [5,6]. Genetic studies pay great attention to the factors that affect both LS and successful aging, which scientists understand as the absence of chronic diseases and the ability to function effectively at physiological and psychological levels [7,8,9,10]. However, LS is determined by the complex interaction of many factors (from genetic to numerous environmental factors) [11]. Basic postgenomic “omics” sciences, such as transcriptomics, proteomics, and metabolomics, can provide additional information about changes in the body at “postgenome” molecular levels (from transcripts to low-molecular-weight substances) [12]. At the same time, metabolomics occupies a special place in scientific studies, since the metabolome, being the end point of all biological events occurring in the body, can provide information about all realized in the organism’s molecular mechanisms of aging. The metabolome is formed by metabolites, low-molecular-weight substances that are substrates, intermediates, and products of biochemical reactions occurring in the body. Metabolites play a key role in energy production, and signal transmission, carry information about the state of the body and ongoing processes, and therefore can be biomarkers of aging or be an integral part of the metabolic signature that reflects the state of the whole organism in the aging process.

## 2. Theories of Aging

Considering metabolomics studies of aging, first of all, it is necessary to mention the theories of aging associated with metabolites, such as the free radical theory and the calorie restriction theory [4,13,14].

Among the oldest theories of aging is the free radical theory. It was first proposed by Denham Harman in the 1950s [4,15]. According to this theory, free radicals pose the greatest danger to DNA. If DNA is irreversibly damaged, this has serious consequences for human health, as illustrated by several genetic disorders of DNA repair, all of which lead to the manifestation of signs of premature aging [16]. Damage to large molecules by free radicals is known as oxidative stress, which was proposed by Harman as a cause of aging and later as a factor in chronic inflammation. In the 1970s, Harman assumed the key role of mitochondria in the formation of free radicals that damage cells and proposed the mitochondrial theory of aging [17]. According to this theory, aging is due to the cumulative effects of free radicals on mitochondrial DNA. Initially, the theory was largely not supported by the scientific community. It gained recognition only with the discovery of superoxide dismutase (SOD, an enzyme that decomposes the superoxide radical) and hydrogen peroxide produced by mitochondria. Later, in support of the mitochondrial theory of aging, studies appeared demonstrating that the ectopic expression of “radical scavengers”, such as catalase and SOD, contributes to an increase in LS in experimental models [18,19]. In addition, genetic manipulations to increase LS are accompanied by an increase in the level of antioxidants, for example, in mice [20].

The mitochondrial theory of aging has been questioned since around 2005. Recent genetic studies show that the LS of the nematodes *Caenorhabditis* and *Drosophila* is increased due to the partial inactivation of mitochondrial SOD, the mitochondrial complex proteins, and mitochondrial ribosomal proteins [21]. Moreover, it was observed that low levels of reactive oxygen species can improve systemic adaptive mechanisms (“mitohormesis” [22]) and increase the LS of nematodes. It should be noted that, according to new data, the hypothesis has been put forward that mitohormesis can occur in mammalian macrophages [23].

The calorie restriction theory suggests that calorie restriction (CR) or intermittent fasting (IF) effectively increases the LS of model animals. When caloric intake is reduced by 20–30%, LS increases by 20% or more in animals such as mice, flies, fishes, and spiders [4,24]. During CR, a decrease in oxidative stress is observed, which indicates its role in this theory [25]. During CR, several signaling molecules are activated or inactivated, such as sirtuin, TOR kinase, and AMP-activated protein kinase. Among the targets for these signals is the transcription factor FoxO of the Fox family (Forkhead box), which activates a group of radical scavenger genes and acts as a tumor suppressor [26,27,28,29]. The transcription factor FoxO is also involved in the regulation of proliferation, cell differentiation, apoptosis, cellular response to stress, as well as aging, and LS [30]. A recent study suggested that FoxO modulation through signaling molecules induces the elimination of senescent cells in the body [31]. Interestingly, chemical compounds that mimic CR conditions are effective both in extending the LS of the organism in experimental models and in treating diseases associated with aging: resveratrol (sirtuin activator) against obesity, rapamycin (TOR-kinase inhibitor) as an antitumor drug or immunosuppressant, and metformin (AMP-activated protein kinase activator) in the treatment of diabetes [32,33,34,35].

Another sirtuin activator is NAD+, a classical coenzyme mediating many redox reactions, whose participation in antiaging therapy has been actively discussed [36,37]. NAD+ plays an important role in the aging process as it participates in various pathways of energy metabolism. The level of NAD+ decreases with aging, leading to many age-related pathologies. Restoring NAD+ by adding intermediates such as nicotinamide mononucleotide (NMN) can greatly facilitate age-related functional decline [38].

Little is known about the effect of CR or fasting on human LS. Some epidemiological studies show that slightly overweight people live longer [39]. In addition to CR, supplementation of food with some metabolites, such as NMN [40], branched-chain amino acid (BCAA) [41], antioxidants, beta-carotene, vitamin A, or vitamin E [42], is being studied as an interventional approach against human aging.

Thus, theories of aging indicate that aging is accompanied by systemic metabolic changes. In recent decades of studying aging, numerous enzymes and metabolites have been discovered that belong to different metabolic pathways and are involved in the regulation of aging [43]. Their important role is known in disorders of protein homeostasis, the nutrient recognition system (the insulin signaling pathway, mTOR, AMP-activated protein kinase, and sirtuin signaling pathways), and in mitochondrial dysfunction. However, there is still no complete picture of the interaction between metabolic processes and aging. To expand our knowledge of the mechanisms of longevity, methods for simultaneously measuring many metabolites, such as untargeted metabolomics methods, are best suited.

## 3. Approaches for Metabolomic Profiling

Metabolomics is the youngest and fastest developing among the “omics” sciences, which makes it possible to obtain a picture of the current metabolic status of the body associated with physiological and pathophysiological processes [44,45]. The subject of metabolomics research is numerous low-molecular-weight substances, both of endogenous and exogenous origin. They are participants in metabolic pathways that can serve as biomarkers indicating various physiological and/or pathological conditions of the body [4,46]. Moreover, the metabolome is the final point of cascades of biological events resulting from the complex interaction of genes, proteins, biochemical reactions, and environmental factors [47], which makes metabolic profiles a source of new data for hypotheses of the molecular mechanisms of aging [43,48,49,50,51,52,53].

In recent years, panoramic profiling of metabolites has become an effective tool for studying biological processes associated with aging [44,49]. Metabolomic profiling is a new method aimed at the simultaneous measurement of a large number of low-molecular-weight substances in biological samples [54,55,56]. The strength of metabolite profiling lies in its untargeted nature, which makes it possible to reveal new knowledge by tracking changes in the whole variety of metabolites [51]. In addition, untargeted metabolite profiling is potentially clinically applicable, namely, to monitor the aging processes, as well as to implement antiaging therapies [57,58].

Information about hundreds or even thousands of metabolites in a biological sample in a single analysis is provided by nuclear magnetic resonance (NMR) spectroscopy [59,60] and mass spectrometry (MS), which can be combined with high-performance liquid chromatography (LC) or gas chromatography (GC) [61,62,63]. Mass spectrometers make it possible to analyze hundreds or thousands of metabolites in a sample in pico- and femtomole concentrations, both after preliminary separation of the sample substances using chromatography or electrophoresis, and without separation by direct infusion mass spectrometry (DIMS), which involves the direct introduction of the analyzed biomaterial into the ionization source of the mass spectrometer [64]. This makes MS the main analytical tool in metabolomics [63] for panoramic analysis, when it is necessary to obtain data on a variety of metabolites belonging to various chemical classes and metabolic pathways.

As a result of the use of MS, a large array of data is obtained that requires further bioinformatic processing to identify the necessary information [65]. First of all, MS data are subjected to standardization or normalization [66,67]. The choice of a further method for data processing directly depends on the purpose of the study [68]. The principal component analysis (PCA) or the independent component analysis (ICA) are used to reduce the dimension of the data. PCA is preferred for metabolomic analysis and can be performed by most statistical programs [69,70]. ICA is used in metabolomics when the choice of a component is not so critical, since it allows one to ignore the technical variability of mass spectra obtained with different instruments [71]. It is also possible to use several methods together [72,73].

Sample classification usually requires the use of cluster analysis. To identify biomarkers, methods are used that work on groups of samples with predetermined parameters (for example, whether samples belong to subjects of different ages) [74]. Among the most commonly used methods for assessing the diagnostic or prognostic power of molecular biomarkers is the Area Under Receiver-Operator Characteristic Curve (AUC) [75]. When AUC is greater than 0.5, the biomarker exhibits diagnostic or prognostic properties that increase as AUC approaches 1 (corresponding to 100% diagnostic sensitivity and specificity).

Due to the growing number of metabolomic studies, the development of special software for the analysis of metabolite spectra (for example, MET-IDEA [76], MathDAMP [77], and TagFinder [78]) has become topical. Many manufacturers of mass spectrometric equipment offer their software packages for the analysis of metabolomic data. For example, the commercial software Metabolic Profiler (Bruker Daltronics, Billerica, MA, USA) allows pre-processing of the resulting mass spectrometric metabolomic data and comparative analysis of metabolite profiles.

In addition to identifying biomarkers related to the aging process, the analysis of the resulting metabolic profiles allows for a systematic analysis of changes in metabolic pathways. This analysis has been made possible by the development of several databases of metabolomes of various organisms and specific biological fluids. Metabolite annotations in such databases include chemical composition, metabolite detection method, concentration data for normal and pathological conditions, as well as information about metabolic pathways in which metabolites are involved. Among most well-known databases, there is HMDB (Human Metabolome Database) (https://hmdb.ca; accessed on 18 September 2022) is an open-access database containing detailed data (chemical, clinical and biochemical information) on more than 40,000 metabolites that have already been identified or most likely can be found in the human body. There are also several popular databases of metabolites for different organisms, including the KEGG (Kyoto Encyclopedia of Genes and Genomes) (https://www.genome.jp/kegg; accessed on 18 September 2022), MetaboLights (https://www.ebi.ac.uk/metabolights; accessed on 18 September 2022), LipidMaps (https://www.lipidmaps.org; accessed on 18 September 2022), and Metlin [79]. The currently known metabolic changes associated with age are in the MetaboAgeDB database [80].

**Figure 1:**
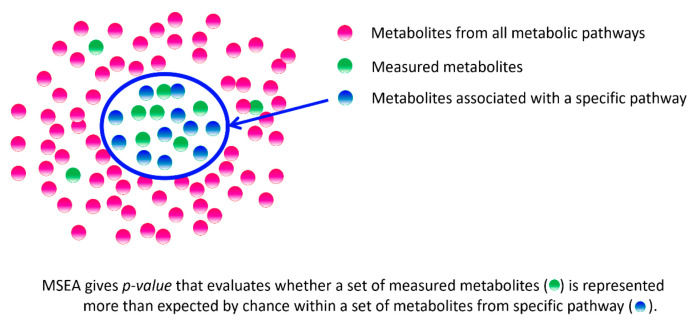
Principle of metabolite set enrichment analysis (MSEA).

The projection of measured sets of metabolites onto metabolic pathways is among the popular and widely used analyses, which makes it possible to identify biological insights at the level of metabolic pathways. Among the common ways to do this is metabolite set enrichment analysis (MSEA), which allows obtaining a statistical estimate of such projection [81]. MSEA is conceptually similar to a widely used genetics tool, known as gene set enrichment analysis (GSEA), and, in general, MSEA gives the probability that the measured metabolites correspond to a certain metabolite set, for example, a specific metabolic pathway (Figure 1).

## 4. Untargeted Metabolomic Profiling in the Study of Aging in Animal Models

Aging is a fundamental biological process whose mechanism is still largely unknown due to its complexity and multifactorial nature. Animal models simplify the study, so a significant amount of knowledge has been gained from such studies [82]. The complex interactions between factors that influence aging and LS, and genes that influence longevity are easier to study in short-lived, simpler organisms. Yeasts, worms, fruit flies, or mammalian models such as mice, dogs, and monkeys have already helped shed light on the aging process [83]. Based on genetic studies in animal models, several mechanisms of aging associated with metabolism became known [84,85,86,87].

Even though lower organisms are not a directly suitable model for the study of biological processes and diseases in mammals, they are nevertheless widely used as an effective model for elucidating the molecular basis of aging. Many intracellular and intercellular signaling pathways, as well as molecular interactions between body tissues, have a high degree of homology even among evolutionarily distant organisms [88]. The main advantages and disadvantages of model organisms are presented in Table 1.

The advantages of animal models are cost reduction, ease of maintenance of the studied organisms, and the possibility of modeling experiments in a laboratory [91,92,93]. The short LS of simple organisms is another key advantage in aging research, allowing both parallel and serial experiments to be carried out within a reasonable time frame [83]. Certainly, a direct extrapolation of the biological mechanisms found in invertebrates, fishes, etc., on mammalian organisms may not be entirely correct, but it sheds light on the diversity of molecular bases of LS and longevity that exists in nature.

**Figure 2:**
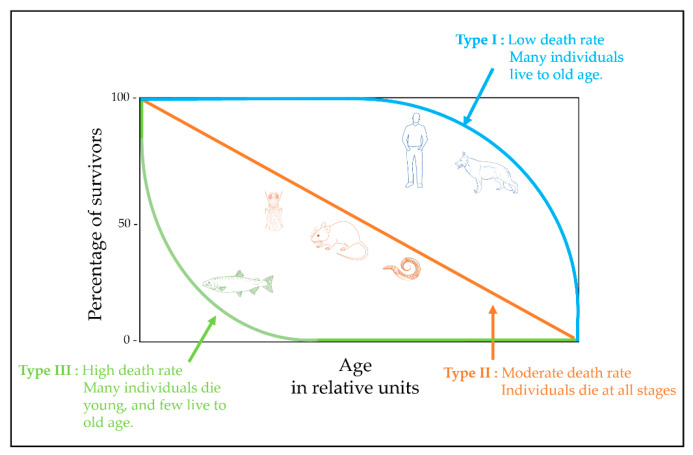
Survivorship curves. Adapted from Holtze S. et al. [96].

In the process of evolution, under the influence of external factors, different types of animals formed different strategies for survival, which is reflected in their survival curves (Figure 2). The study of species with different survival strategies certainly expands our understanding of the aging process. The difference in strategy is usually taken into account when designing a study and interpreting the results. For example, *Drosophila* relates to species with a type II survival curve in which the proportion of living organisms falls almost linearly over time. Throughout life, the chances of dying due to predators, diseases, accidents, etc., are usually constant. Therefore, fly samples for the study of aging can be obtained throughout life [94]. A human belongs to the type I survival curve, in which the proportion of people is high in early and middle age and decreases as a human approaches old age, and mortality is maximum in old age. Therefore, cohorts for the study are formed with this fact in mind, for example, a group of older people versus young people in a comparative study [95].

Regarding metabolomics, several studies of aging were performed in animal models, both mammalian (mice, rats, dogs) [62,82,97,98] and non-mammalian (vertebrate and invertebrate) [94,99,100,101,102,103,104,105] (Table 2). As a rule, early works are an integral part of complex studies, which include, in addition to metabolomics, histological, biochemical, and genetic studies.

Thus, for studying aging, there are different model organisms, with their own advantages and disadvantages (Table 1). Some are simple for modeling experiments, but evolutionarily far from humans, others are close in biology to humans but difficult to research. All this together justifies the existence of various models. However, the difference in models leads to different designs for untargeted metabolomic studies, including sampling timing, sample preparation protocols, and metabolite measurement method (Table 2). To review the available data and, as a result, to draw general conclusions about the role of metabolites in the aging process, the results obtained on different animal models—from worms to humans, are further considered.

## 5. Metabolomic Profiling of *Caenorhabditis elegans*

Due to the short LS and ease of cultivation in the laboratory, the nematode *Caenorhabditis elegans* is among the most popular model organisms to investigate the molecular mechanisms of aging. Other advantages of *C. elegans* include the fully sequenced genome, the ease of genetic intervention through bacteria-feeding, and the research-proven effects of altering either temperature or the amount of food on LS changing [115].

In the research of Copes et al., a global assessment of metabolite levels in young and old nematodes was carried out using GC–MS and led to the successful identification of 186 metabolites [99]. The data were analyzed using PCA, which showed that most of the changes in metabolite levels were due to age differences. Metabolomic analysis results showed that aged *C. elegans* had reduced levels of purine and pyrimidine metabolites, reduced levels of free hydrophobic amino acids, altered S-adenosylmethionine metabolism, elevated sorbitol, elevated free fatty acid levels, and a shift in cellular redox balance. Moreover, it was found that with age, the pathway of amino acid biosynthesis changes the most, primarily due to a significant degree of overlap of this pathway with other metabolic processes. Of the 24 metabolites identified in this pathway, 14 were significantly altered with age, including 7 amino acids. Of the 14 metabolites that were altered, 11 were significantly reduced in older nematodes.

Wan et al. used *C. elegans* hermaphrodites as a model to study changes in metabolic pathways during aging and how deletion of germline stem cells (GSCs) leads to infertility and increased LS in long-lived glp-1 mutants [100]. Metabolomic profiling was performed by combining NMR and LC–MS. Analysis was performed using PCA, hierarchical, and supervised orthogonal projection to latent structure with discriminant analysis (OPLS-DA). The results showed that aging is accompanied by metabolome remodeling. Age-changing metabolic pathways included glutathione metabolism, glutamate metabolism, purine and pyrimidine metabolism, taurine and hypotaurine metabolism, and the tricarboxylic acid cycle (TCA cycle). Analysis of the metabolic profiles of long-lived glp-1 mutants showed that glp-1 mutants regulate the levels of many age-related metabolites to delay aging, including an increased level of intermediate products of pyrimidine and purine metabolism and reduced levels of TCA cycle intermediates.

Gao et al. also used *C. elegans* as a model to analyze age-related changes in the metabolome and analyzed metabolite profiles throughout life, including larval development, reproductive phase, and aging [101]. Metabolomic analysis was performed by LC–MS/MS, which allowed the detection of more than 600 metabolites. Marked changes were observed in the levels of fatty acids, amino acids, and phospholipids throughout the life of the worms. A dramatic shift in lipid metabolism was observed after early adulthood. The highest levels of most amino acids occurred during development, except aspartic acid and glycine, which increased in aging worms.

## 6. Metabolomic Profiling of *Drosophila*

*Drosophila* is among the most widely used model organisms for various biomedical studies. The high homology of intracellular processes with processes in mammalian cells makes fruit flies an attractive tool for research in cell biology, genetics, and in the study of human diseases.

*Drosophila* is a powerful experimental model for testing hypotheses about biomarkers of aging that was convincingly shown by Zhao et al. (2022) by observing adult cohorts of 20 *Drosophila* Genetic Reference Panel (DGRP) strains selected to represent the breadth of LS variation [104]. By comparing LS and age-related functional traits (fertility and activity) with metabolic profiles obtained using LC–MS, it was shown that the metabolome is a biological clock that predicts not only the age of flies but also LS. Targeted analysis revealed two pathways that are highly represented among the features associated with LS. First, they identified metabolites associated with tryptophan/kynurenine metabolism. The second group of metabolites includes the amino acids arginine, ornithine, and proline, as well as their related metabolites.

In the research of Maslov et al. (2021), the identification of signs of longevity was based on a comparative study of the metabolomic composition of twelve *Drosophila* species with different LS (*D.virilis*, *D.ananassae*, *D.saltans*, *D.simulans*, *D.austrosaltans*, *D.bipectinata*, *D.yakuba*, *D.melanogaster*, *D.willistoni*, *D.erecta*, *D.kikkawai,* and *D.Biarmipes*) [94]. The studied species have an identical body structure and life cycle [116], at the same time, as a result of a long evolution, they have acquired significant phenotypic differences (size, weight, etc.) [117]. As an assessment of the degree of aging, relative age was proposed, which was expressed as a percentage of the maximum LS of the species. Thus, samples of equal relative age from each species were selected and combined into cohorts for comparative analysis (long-lived species, medium-lived species, and short-lived species) [94]. DIMS was used for metabolomic profiling.

The results of a comparative analysis showed a significant difference in metabolites belonging to different chemical classes: carbohydrates, amino acids, carnitines, biogenic amines, and lipids [94]. The highest level of differences in metabolites was observed in long-lived species. It was suggested that this level may be due to the up-regulation of pathways involving these metabolites. Metabolite set enrichment analysis (MSEA) of metabolic pathways revealed seven involved metabolic pathways: aminoacyl-tRNA biosynthesis, valine, leucine, and isoleucine biosynthesis, arginine biosynthesis, arginine and proline metabolism, alanine, aspartate, and glutamate metabolism, glycine, serine, and threonine metabolism, and starch and sucrose metabolism [94]. Thus, a comparative analysis of the metabolic composition made it possible to determine the biological pathways that evolved between closely related species, and thus suggest that some of these pathways may be associated with a fast or slow rate of development of age-related processes.

Avanesov et al. (2014) used untargeted LC–MS for metabolomic profiling to study age-related changes of >15,000 metabolites in *D.melanogaster* males on a control diet and on a restricted diet that increased LS [51]. It was shown that with age there is an increase in the types of age-associated metabolites, which presumably indicates a cumulative effect, as a result of which multiple damages can have an additive effect on the LS. It is noteworthy that the number of detected compounds levels off at the end of life, and this pattern is associated with survival. The authors conclude that aging is characterized by a gradual remodeling of the metabolome, and the slowdown in this remodeling is associated with molecular damage and LS [51].

Hoffman et al. (2014) describe the effect of age, sex, genotype, and their interaction on the metabolomic profiles obtained using LC–MS for 15 *D.melanogaster* inbred lines [50]. Of all the metabolites analyzed, more than a quarter was significantly associated with age, sex, genotype, or their interaction, and multivariate analysis showed that individual metabolomic profiles for these features are highly predictable. Using MSEA, metabolic pathways associated with age, sex, and genotype were identified, including pathways associated with the metabolism of carbohydrates and glycerophospholipids, neurotransmitters, amino acids, and the carnitine shuttle [50]. The results showed that metabolomic profiles can reveal both the mechanisms of aging and their relationship with genotype and sex.

In the study of Laye et al. (2015), the same group of researchers, as in the previous work, analyzed tissue samples on an LC–MS platform with double column chromatography (reverse phase and ion exchange columns) [106]. Changes in the metabolome of the head, thorax, and abdomen were studied in fruit flies of different ages fed either a nutrient-rich diet ad libitum (AL) or a nutrient-restricted diet (DR). The multivariate analysis separated the metabolome by diet, different tissues, and age. DR significantly changed the metabolome and, in particular, slowed down the age-related changes in the metabolome, preventing a decrease in the stability of homeostasis during aging. MSEA allowed identifying several known (e.g., amino acid and NAD+ metabolism) and novel metabolic pathways that are involved in DR influencing aging [106].

Avanesov’s results, similar to those of Laye, show that a diet that increases LS “shifts” both the transcriptome and the metabolome towards a “younger state” [51,106]. Other similarities include age separation using principal component analysis and the identification of similar but not identical sets of metabolites. However, direct comparisons between the two studies should be made with caution, as there are several important methodological differences between the studies (including different mass spectrometry protocols and different fly strains). Not to mention, a non-targeted metabolic analysis of flies was performed by Laye et al. on specific tissues compared to the study by Avanesov et al. where whole flies were used.

Wang et al. demonstrated a new untargeted MetTracer metabolomics technology for isotope tracking using LC–MS analysis [105]. This technology allows traceability of labeled metabolites across the entire metabolome, providing monitoring of metabolic activity during aging and facilitating understanding of metabolic regulation in living organisms at the system level. Using *Drosophila* as a model organism, changes in metabolic activity were found during aging. Metabolic pathways associated with carbohydrate metabolism were enriched in metabolites with reduced metabolic activity during aging. In contrast, metabolic pathways associated with amino acids and purine metabolism were enriched with metabolites with increased metabolic activity during aging. In addition, a metabolic shift from glycolysis to serine metabolism and purine metabolism was found as *Drosophila* ages. An important conclusion was made in the work about the disturbance of metabolic coordination between the three metabolic pathways in the tissues of the head and muscle tissue, as well as between tissues, and such disturbance contributes to the aging of *Drosophila*.

## 7. Metabolomic Profiling of Fishes

Fishes are a promising model for studying the biochemical foundations of aging by comparative analysis. The existence of fish species with different types of aging makes it possible to combine several species into analyzed groups and thus successfully exclude species-specific variability from the analysis [103]. Another advantage of this experimental model is the possibility of projecting the results of the analysis onto the processes occurring in mammals since most fish organs are similar to those of other vertebrates [103].

A metabolomic study of the blood plasma of three groups of predatory fishes with different aging rates was carried out at the Institute of Biomedical Chemistry (Moscow, Russia) [102,103]. The first group included long-lived fish species (pike (*Esox Lucius*) and sterlet (*Acipenser ruthenus*)), and the second group included species with gradual aging, the same as observed in many mammalian species of similar size (zander (*Sandra lucioperca*) and perch (*Perca fluviatilis*)) and the third group—species with a very short life cycle (salmon (*Oncorhynchus keta*) and pink salmon (*Oncorhynchus gorbuscha*)) [118]. All studied fishes were at the adult stage, and the studied groups included fishes both before and after spawning. Metabolite profiling by DIMS revealed a set of metabolites whose plasma levels are associated with the rate of aging [102]. The results of this study demonstrate that the profiles of blood plasma metabolites in fishes with different aging rates differ, and the revealed differences are largely associated with the rate of aging, which does not depend on the fish species. It was shown that 23 metabolites are associated with the rate of aging, 15 of them are dipeptides, di- and triglycerides, fatty acids, phosphoethanolamines, and phosphatidylcholines. The data obtained are consistent with the already known pathophysiological mechanisms of aging and the results of previous studies [102].

Another study conducted by the same researchers focused on the analysis of the metabolic composition of the tissues of the skeletal muscles of fish with different aging rates [103]. Metabolomic profiling was performed by DIMS. Multivariate analysis revealed about 80 group-specific metabolites related to amino acids, lipids, biogenic amines, as well as intermediates of glycolysis, glycogenolysis, and the citric acid cycle, which have undergone changes and are possibly involved in biochemical pathways related to aging [103]. Based on the results, the authors conclude that the power of antioxidant protection, the productivity of anabolic processes, and, possibly, the efficiency of energy metabolism in skeletal muscles are associated with the fish LS. A decrease in the intensity of these processes or their damage can lead to the loss of muscle mass and strength with age [103].

## 8. Metabolomic Profiling of Rodents

Quite often, metabolite profiling was used to study the signs of aging in mice [62,108], identifying a set of markers that confirmed that aging is associated with changes in nutrient sensitivity, lipid, and amino acid metabolism, and redox homeostasis.

Houtkooper et al. (2011) characterized clinical, biochemical, and metabolic changes in young mice as compared with aged mice, contributing to the determination of the aging phenotype [62]. C57BL/6J mice were used as they are particularly suitable for the study of metabolic disorders. In this study, untargeted tissue metabolomics complemented biochemical and histological data, in vivo phenotyping, and targeted metabolomics data. Using GC–MS and LC–MS/MS to detect metabolic changes in aging muscle and liver tissues, a set of metabolites that change with aging, including those involved in fatty acid and glucose metabolism, was identified. Cross-validation of established pathways by different approaches (detection of metabolites in metabolomic studies coupled with gene expression analysis) enhanced the potential value of metabolites as biomarkers and provided high accuracy in the identification of molecular and biochemical profiles of aging.

Glucose, as well as intermediate products of glycolysis and glycogen metabolism, such as maltose and maltotetraose, were found in the liver and muscle as biomarkers of aging. In both liver and muscle, accumulation of glycogen intermediates suggests an alteration in glycogen metabolism in aged mice, while elevated levels of lactate and reduced glycolytic intermediates suggest increased anaerobic glycolysis. Changes in glucose and glycogen metabolism were also indicated by increased levels of glucose, glucose-6-phosphate, and maltose in muscles since the level of maltose in muscles constantly increases with increased glycogenolysis [62].

Metabolite profiling can be used not only to determine the metabolic status of an organism under conditions of interest but also for the molecular phenotyping of organisms with mutant genotypes. Tomás-Loba et al. (2013) determined the serum metabolite profile of 117 male and female wild-type mice with different genomes at the age of 8 to 129 weeks on the LC–MS platform, which made it possible to isolate a metabolic characteristic that reliably and accurately predicts their age [108]. The overall profile of metabolites was used in a multivariate predictive model based on the projection to latent structures (projection to latent structure, PLS), resulting in a reliable metabolomic model of aging in wild-type mice. 48 biomarkers were identified for which there is a significant correlation with age. Biomarkers included phospholipids, fatty, and other organic acids, and this is consistent with the fact that the extraction methods provided the maximum coverage of these families of compounds [108]. In addition to lipids, age-related biomarkers included other molecules such as creatine, methionine, and uric acid. It remains an open question whether these biomarkers play a role in aging or are the result of secondary events such as age-related diseases or muscle loss [108].

Since there are many causal relationships between aging and DNA damage repair deficiency, Nevedomskaya et al. (2010) studied ERCC1d/- mutant mice, which have a modified ERCC1 gene involved in DNA repair after nucleotide deletion, as a result of which the animals have a premature aging phenotype [82]. Profiling of metabolites in the blood serum and urine of mutant and wild-type mice was performed using 1H NMR spectroscopy. Metabolomic profiles of mice aged 8–20 weeks were submitted to principal components (PCA) and discriminant analysis by the method of Partial Least Squares (PLS-DA). The metabolomic profiles of mutant and wild-type mice were similar at a younger age, and the difference between them became more noticeable with age. This fact indicated that ERCC1d/- mutants develop more or less normally until puberty, but begin to show accelerated senescence after they reach maturity and therefore they represent a model for studying the aging process [82]. The main differences between mutant and wild-type animals were associated with changes in lipid and energy metabolism, transition to ketosis, and decreased liver and kidney function. Moreover, most of the differences in serum between wild-type and mutant animals were associated with changes in the levels of various lipids in ERCC1d/- mutants compared with wild-type mice. Low-density lipoproteins (LDL) and very low-density lipoproteins (VLDL) decreased, while high-density lipoproteins (HDL) increased [82]. These changes resemble the pattern in blood lipoprotein composition in a state of caloric restriction [119]. NMR analysis also showed that in the serum of ERCC1d/- mutants, compared with wild-type mice, the levels of glucose and lactate are reduced [82], indicating that a molecular phenotype is associated with CR. The authors of the work suggest that in ERCC1d/- mice, a specific “survival” reaction of the body is activated, similar to that during CR, which primarily affects energy metabolism and leads to ketosis [120].

Recently, a group of scientists from China used untargeted LC–MS to detect changes in metabolites in liver tissues and serum in young and old rats, including those after liver transplantation of young animals [109]. This was done to understand the mechanisms underlying liver aging, which not only impairs organ function but also systemically harms the body’s metabolism. A total of 153 liver metabolites and 83 serum metabolites differed between young and aged non-transplanted rats. Among these metabolites, 7 were observed in both the liver and serum. Five weeks after the transplantation of the young animal liver, the levels of 25 metabolites in the transplanted liver were similar to those in the liver of aged recipients, which was probably the result of the influence of the body of aged animals on the graft. Among these metabolites were organic acids and their derivatives, lipids, and lipid-like molecules. Metabolite analysis revealed nine metabolic pathways including glycerophospholipids, arachidonic acid, histidine, and linoleate. Thus, this study has identified important metabolites and metabolic pathways associated with age, as well as the interaction between the liver and the internal environment of the body.

## 9. Metabolomic Profiling of Dogs

Dogs are not among the most popular models for studying aging, but we have included the studies described below nonetheless since these studies allowed us to perform untargeted metabolomic profiling of large cohorts of pets of different ages.

The aim of the study by Wang et al. (2007) was to investigate the lifetime metabolic changes in urine in control feeding (CF) or diet-restriction (RD) dogs as long-term caloric restriction without malnutrition was shown to prolong life and slow age-related morbidity [98]. 1H NMR spectroscopy was used to monitor the metabolomic profiles of urine samples. Changes in metabolites in both groups (CF and RD) followed the same trajectory, suggesting that age-related changes predominate in the metabolic profiles of urine, with aging having a greater effect on metabolism than dietary restriction. Thus, with age, an increase in creatinine excretion with urine is observed, reaching a maximum at the age of 5 to 9 years and subsequently decreasing in parallel with a decrease in lean body mass [98]. In addition, diet-related metabolic changes were also characterized. Metabolites associated with energy metabolism, such as creatine, 1-methylnicotinamide, lactate, acetate, and succinate, were reduced in the urine of dogs with RD. Both aging and dietary restriction changed the activity of the intestinal microflora, which was manifested in the level of aromatic metabolites and aliphatic amine compounds. This analysis allowed to track the metabolic response to two different physiological processes throughout the life of the dogs and gain a more general idea of the increase in the LS of higher mammals [98].

A group from the University of Helsinki (Finland) analyzed 2068 blood serum samples from healthy domestic dogs of 22 different breeds using untargeted metabolomics based on NMR spectroscopy [110]. Using generalized linear models, age, breed, sex, neutering, diet type, and fasting time were found to significantly affect metabolite profiles in dogs. In particular, age caused the most significant differences in metabolite concentrations, affecting 112 of the 119 metabolites measured. Moreover, the levels of most of them increased with age, and 21 of 119, mainly lipids and GlycA (a marker of protein glycosylation), even exceeded the upper limit in dogs older than 14 years. GlycA levels may be elevated due to subclinical inflammatory processes that are relatively common in older dogs [121]. However, changes in the immune status during aging can also lead to chronic low-level inflammation, potentially increasing the concentration of GlycA [121]. Almost all cholesterols, triglycerides, and lipoproteins showed the highest levels in older dogs, indicating age-related changes in lipid metabolism [122].

## 10. Untargeted Metabolomic Profiling in the Study of Human Aging

Compared to model organisms, the study of human aging initially requires a different approach than working with model organisms. The reasons for this are ethical restrictions, difficulties in setting up experiments, the complexity and variability of the aging process itself in humans (the so-called “diversity of aging”) (Table 1). Therefore, untargeted metabolomic profiling comes to the fore in humans related studies (Table 2).

Lawton et al. (2008) analyzed changes in the human plasma metabolome with age in an age- and a sex-balanced cohort of 269 people [111]. A metabolomic analysis using GC- and LC–MS was performed on more than 300 metabolites. In 100 of them, the change in concentration was associated with age. Much fewer metabolites reflected differences in sex and race. With age, changes in protein, energy, and lipid metabolism, as well as changes associated with oxidative stress, were observed. The levels of tricarboxylic acid intermediates, creatine, essential and non-essential amino acids, urea, ornithine, polyamines, and markers of oxidative stress (e.g., oxoproline, hippurate) increased with age. The levels of compounds associated with lipid metabolism, including fatty acids, carnitine, β-hydroxybutyrate, and cholesterol, were lower in the blood of young people. Relative concentrations of dehydroepiandrosterone sulfate (a putative antiaging androgen) were lowest in the oldest age group. The observed increase in blood concentrations of some xenobiotics (for example, caffeine) in the blood of elderly people may reflect a decrease in the activity of cytochrome P450 in the liver [111].

A group of scientists from Japan developed the method for analyzing whole blood, plasma, and red blood cells [95]. Using LC–MS, an untargeted metabolomic blood analysis was performed on 15 young (mean age 29 years) and 15 elderly (mean age 81 years) individuals [95]. 14 blood metabolites have been identified that increase or decrease markedly with age: 1,5-anhydroglucitol, dimethylguanosine, acetylcarnosine, carnosine, ophthalmate, UDP-acetylglucosamine, N-acetylarginine, N6-acetyllisine, pantothenate, citrulline, leucine, isoleucine, NAD+, and NADP+. Six of these are enriched in erythrocytes, suggesting that erythrocyte metabolomics is valuable for human aging research. Age differences are partly explained by reduced antioxidant production or slower urea metabolism in the elderly. Additional analysis showed that some age-related compounds correlate with each other, suggesting that aging affects them simultaneously [95].

Recently, a group from Stanford University (California, USA) published a study covering the human age from 6 months to 82 years, in which they performed an untargeted metabolomic analysis of blood plasma with a quantitative determination of 770 metabolites in a cohort of 268 healthy people, including 125 pairs of twins [58]. LC–MS was used, including separation by complementary Hydrophilic Interaction Liquid Chromatography (HILIC) and Reversed-Phase Liquid Chromatography (RPLC). Cluster analysis, machine learning, and metabolic pathway analysis were used to describe the trajectories of changes in metabolite concentrations throughout life and to detect metabolic pathways that are disrupted with age [123]. Six major aging trajectories were identified, some of which were linked to key metabolic pathways such as progestin steroids, xanthine, and long-chain fatty acid metabolism. Machine learning models were successful in predicting age and, in combination with metabolic pathway analysis, were used to study the biological processes of healthy aging. The models identified metabolites previously described in aging processes, such as steroids, amino acids, and free fatty acids, as well as new metabolites and pathways. Interestingly, the metabolic profiles of twins become more dissimilar with age, suggesting a non-genetic, age-related variability in metabolic profiles in response to environmental exposure.

A more extensive study in terms of the number of samples was carried out by another group of American scientists from the University of Wisconsin (Madison, USA) [112]. In a longitudinal metabolomic study of age and sex, plasma samples from the Wisconsin Registry for Alzheimer’s Prevention (WRAP) were used. The cohort included participants who did not have dementia at the time of inclusion. Metabolomic profiles were obtained from 2344 fasting plasma samples from 1212 participants at various time intervals. Of the 1097 metabolites tested, 623 (56.8%) were associated with age and 695 (63.4%) with sex. It was shown that aging affects plasma levels of most metabolites with a broader effect on metabolites in women than in men. Approximately twice as many metabolites were associated with age in a stratified analysis of women compared to men. The 68 metabolites differed significantly by sex, primarily including sphingolipids, which tended to increase in women and decrease in men with age [112]. Differences in plasma lipid steroid levels, including androgens, progestins, and pregnenolone, were most significant for both age and sex. Additionally, whole-genome genotyping suggested that many metabolites are strongly influenced by a combination of genomic and environmental factors [112].

The results of the study with the WRAP cohort [112], are consistent with the results of earlier cross-sectional studies performed on plasma samples from participants in the TwinsUK study (UK Registry of Adult Twins) [113]. In this study, untargeted MS metabolomic profiling of 1052 serum samples and 5003 plasma samples showed that in a cross-sectional analysis, 165 of 280 (58.9%) tested serum and plasma metabolites were associated with age [113]. This study identified a group of 22 metabolites that correlate with calendar age as well as age-related clinical signs regardless of age. These data illustrate how metabolomic profiling associated with epigenetic studies can identify some of the key molecular mechanisms associated with long-term physiological processes affecting human health and aging.

The German KORA F4 population cohort study, which was also cross-sectional, used data from 1756 fasting serum samples, including 903 women and 853 men [114]. Metabolomic profiling was performed using LC–MS and GC–MS. The results showed that 180 of the 507 (35.5%) tested serum metabolites were sex-linked. In the data from the WRAP cohort study, 98 of these 180 metabolites were present [112], of which 84 were also significantly sex-linked [113]. Among them, there were 33 amino acids (including 11 common amino acids, which were lower in women, except for glycine and serine), 18 lipids (including five long-chain fatty acids and three medium-chain fatty acids, which were higher in women, and three androgenic steroids, which were lower in women) and 18 unknown metabolites (all but one of which were lower in women).

## 11. Summary of Metabolome Profiling Data from Aging Studies

**Figure 3:**
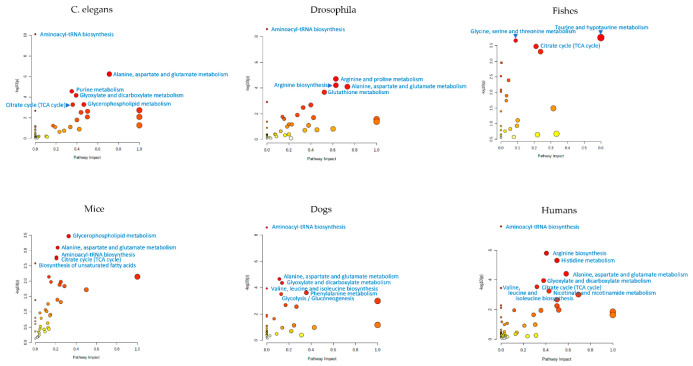
Metabolic pathways in animal and human models potentially involved in the aging processes. Graphs were generated by the metabolite set enrichment analysis (MSEA) using the MetaboAnalyst program (www.metaboanalyst.ca; accessed on 18 September 2022) (“pathway analysis” option) by projecting metabolites onto metabolic pathways. The lists of metabolites associated with aging, which are presented in the papers mentioned in this review, were used for projection. Names of metabolic pathways with statistically significant (*p* < 0.01) enrichment with projected metabolites are shown. The *p* value evaluates whether a measured set of metabolites is represented in the pathway more than expected by chance within a given list of metabolites (*p* values from the pathway enrichment analysis). Metabolic pathways of *Homo sapiens* (for the “Humans” and “Dogs” graphs), *Mus musculus* (for the “Mice” graph), *Danio rerio* (zebrafish) (for the “Fishes” graph), *Drosophila melanogaster* (fruit fly) (for the “Drosophila” graph), *Caenorhabditis elegans* (for the “*C. elegans*” graph) from the KEGG were used to project metabolites. Pathway impact values are from the pathway topology analysis.

When considering metabolome profiling in aging studies and attempting to draw generalized conclusions based on currently available data, it is necessary to take into account the specifics of such studies. The measurement of large sets of metabolites using different objects, different samples, sample preparation protocols, and measurement equipment results in different sets of metabolites being measured in untargeted metabolomic studies. This makes it difficult to generalize findings from different studies. This can be helped by the projection of identified metabolites in various animal models onto metabolic pathways. The best way to do this is to use MSEA to project the lists of aging-associated metabolites presented in this review into metabolic pathways (Figure 3). Even if different metabolites found in different studies participate in the same metabolic pathway, this indicates their participation in identical biochemical processes, and such data will confirm each other and be interpreted in the same way. Figure 3 shows that aging-related metabolites from different animal models are mostly associated with the same metabolic pathways. For example, aminoacyl-tRNA biosynthesis is statistically significantly (at *p* < 0.01) associated with aging in *C. elegans*, Drosophila, dogs, and humans. The citrate cycle is also statistically significantly associated with aging in *C. elegance*, fishes, mice, and humans. This means that the aging processes in the models are very similar to each other, from *C. elegans* to humans. This supports the use of model objects to understand aging processes, including for humans.

**Figure 4:**
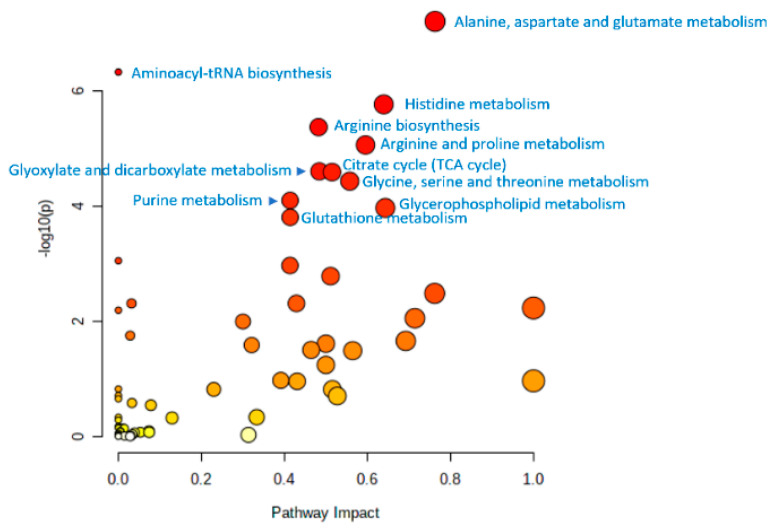
Summary graph of animal and human models for metabolic pathways potentially involved in aging processes. Graph was generated by the metabolite set enrichment analysis (MSEA) using the MetaboAnalyst program (www.metaboanalyst.ca; accessed on 18 September 2022) (“pathway analysis” option) by projecting metabolites onto metabolic pathways. The list of all metabolites associated with aging, which are presented in the papers mentioned in this review, was used for projection. Names of metabolic pathways with statistically significant (*p* < 0.01) enrichment with projected metabolites are shown. The *p* value evaluates whether a measured set of metabolites is represented in the pathway more than expected by chance within a given list of metabolites (*p* values from the pathway enrichment analysis). Metabolic pathways of *Homo sapiens* (KEGG) were used to project metabolites. Pathway impact values are from the pathway topology analysis.

Such an identity allows a generalized conclusion to be drawn from all the results presented in this review, by jointly projecting all metabolites associated with aging onto metabolic pathways (Figure 4). In this case, the data of various models confirm and complement each other, making the generalized conclusion more complete and statistically reliable. Thus, from Figure 4, it can be concluded that the data of untargeted metabolomic studies accumulated to date on different models indicate statistically significantly the involvement in the aging of the 11th metabolic pathways, which are shown in the figure.

## 12. Final Remarks

Living systems have a multilevel organization, which in terms of omics science can be represented as genome > transcriptome > proteome > metabolome. The flow of information in the organism goes from macromolecules—nucleic acids that form the genome and are the most static carrier of information, in the direction of low-molecular-weight substances that form the metabolome—the dynamically changing molecular phenotype of the organism. The metabolome is largely determined by the biochemical reactions occurring in the body, the substrates, intermediates, and products which are metabolites that form the metabolome. At the same time, biochemical reactions are combined into networks—metabolic pathways. Therefore, any changes, including those occurring during aging (genetic instability, epigenetic changes, loss of proteostasis, mitochondrial dysfunction, etc.), are reflected in the molecular phenotype, i.e., in the metabolome, and the analysis of metabolic pathways by measuring the sets of metabolites involved by untargeted metabolomic profiling is a valuable source of information about all the processes involved in aging.

Among the metabolites identified by untargeted metabolomic profiling, known metabolic biomarkers of aging are the first to attract attention [43,95]. For example, among the metabolites identified in humans are 1,5-anhydroglucitol, dimethylguanosine, acetylcarnosine, UDP-acetylglucosamine, NAD+, and NADP+; among the metabolites identified in both humans and mice are carnosine and pantothenate; among the metabolites identified in both humans and Drosophila are N6-acetyllysine and citrulline; among the metabolites identified in human, fishes, dogs, and Drosophila are isoleucine and leucine. This demonstrates that in different animal models, from the simplest to humans, the same metabolites involved in the aging process can be found. Moreover, the projection of metabolites to metabolic pathways showed a statistically significant involvement of the same metabolic pathways in aging. First of all, metabolic pathways related to the metabolism of amino acids, as well as to the metabolism of lipids, purines, and energy metabolism (cycle TCA and glycolysis/gluconeogenesis), certainly reflect processes occurring in any organism during aging—disorders of DNA and protein homeostasis, disorders of nutrient recognition system (the insulin signaling pathway, mTOR, AMP-activated protein kinase, and sirtuin signaling pathways), and mitochondrial dysfunction [43].

Although untargeted metabolomic profiling contributes greatly to determine the involvement of metabolites belonging to different metabolic pathways in the aging processes, there are still many open questions that need to be addressed to complete a picture of the molecular mechanisms associated with aging processes. In addition to the models of aging research described in this review, there are long-lived animals, e.g., as Naked Mole-Rats (*Heterocephalus glaber*) [124], African mole-rats (family *Bathyergidae*) [125], Clownfishes (genus *Amphiprion*) [126], Greenland shark (*Somniosus microcephalus*) [127], which implement different longevity strategies. Untargeted metabolomics profiling of these animals will complete the existing data on the involvement of metabolites in aging.

Among the practical applications of metabolic aging research is the creation of a biological clock. Although a strong association between metabolites and age has been shown, such clocks have not yet been proposed. Possible reasons for this situation include the multifactorial nature of aging, a wide variety of metabolites with high variability in their concentrations, and, as a result, the difficulty of accurately determining age based on metabolomic profile data. The prospects for metabolomics research of aging are associated with this direction.

## 13. Conclusions

Over the years, researchers have used whole-genome sequencing and gene expression data to identify genes associated with aging. However, there is a gap between gene variations and LS. In an attempt to fill this gap, scientists turned to postgenomic technologies, among which metabolomic profiling can be distinguished. Since the metabolome is the end point of cascades of biological events occurring in the body, metabolomic profiling can identify the molecular mechanisms that cause physiological changes that affect human health and aging. The results presented in this review, obtained in studies both in various model organisms and in humans, showed that metabolites that differ significantly in different age groups relate to carbohydrates, amino acids, carnitines, biogenic amines, and lipids. Based on these data, metabolic pathways associated with biological age have been identified, including those related to amino acid, lipid, and energy metabolism. Notably, the aging-associated metabolites identified in different models are largely related to the same metabolic pathways. It is assumed that these data can be used to monitor biological age and predict age-related diseases in the early stages of their development.

## Figures and Tables

**Table 1:** The characteristics of various model organisms for studying aging. Adapted from Allard J.B. et al. [89], Taormina G. et al. [83], and Strange K. [90].

Characteristics	*C. elegans*	*Drosophila*	Fishes			Rodents		Dogs	Humans
			Pacific Salmon	Pike	Carp	Mice	Rats		
Similarity to the human genome	~25%	~50%	>70%	>70%	>70%	~83%	~90%	~85%	100%
Genome size	11/12 chromosomes 21,305 genes	8 chromosomes 14,065 genes	52–74 chromosomes Up to 40,000 genes	18 chromosomes ~22,000 genes	104 chromosomes	40 chromosomes 29,083 genes	42 chromosomes ~25,000 genes	78 chromosomes 36,322 genes	46 chromosomes 63,494 genes
Lifespan ^1^	2–3 weeks	4–6 weeks	Several years	7–10 years	20 years	1–3 years	2–3 years	6–16 years	∼80 years
Age of puberty ^1^	50 h	10 days	2–5 years	3–5 years	2–5 years	9–12 weeks	1.5–3 months	14–18 months	10–16 years
Number of offspring ^1^	300–1400 offspring	∼120 eggs	no more than 20,000 eggs	from 18 000 up to 220 000 eggs	up to 1.5 million eggs	6–12 cubs	8–10 cubs	3–8 cubs	1–2 children
Advantages	- low cost of animals and maintenance - no ethical requirements - short LS - easy to work with - genetically tractable - strains can be archived by cryopreservation	- low cost of animals and maintenance - no ethical requirements - the breadth of LS variation - rapid onset of puberty and high fertility - a wide range of phenotypes - some intracellular processes are similar or homologous to human cells	- vertebrates - no need for ethical requirements - the breadth of LS variation - most intracellular processes and many physiological processes are similar to mammals - high fertility			- mammals - high similarity with the human genome - most cellular processes and physiological processes are similar to humans - available for many genetic manipulations - a wide range of phenotypes - strains archived by cryopreservation of embryos and sperm		- mammals - high similarity with the human genome - most cellular processes and physiological processes are similar to humans	- research is most relevant for improving health
Disadvantages	- relatively simple anatomy - lacks distinct endocrine tissues and various other tissue types - evolutionarily very distant from humans	- strains needed maintain constantly - evolutionarily distant from humans	- evolutionarily distant from humans - long LS			- the expensive cost of animals and maintenance - the need for ethical requirements - relatively long LS - over-reliance on pre-clinical models: many drugs that are effective in mice and rats do not work in humans		- the very expensive cost of animals and maintenance - the need for ethical requirements - long LS	- limited ability to do experiments - the need for ethical requirements - long LS - “diversity of aging”

^1^ mean; LS, lifespan.

**Table 2:** Objects, methods, and main results of the studies presented in this review.

Object of Study	Research Materials	Age of Objects	Profiling Methods	Metabolites and Metabolic Pathways that Change with Aging	References
*C. elegans*	Whole worms	day 4 (young adult), day 10 (the mean length of LS)	GC–MS	Purine and pyrimidine metabolism, free hydrophobic amino acids, S-adenosylmethionine metabolism, sorbitol, free fatty acids, cellular redox balance, amino acid biosynthesis	[99]
	Whole worms	young adult and day 10	NMR spectroscopy, LC–MS	Glutathione metabolism, glutamate metabolism, purine and pyrimidine metabolism, taurine and hypotaurine metabolism, tricarboxylic acid cycle	[100]
	Whole worms	days 1, 3, 5, 7, 9 and 10	LC–MS	Metabolism of fatty acids, amino acids, and phospholipids	[101]
*Drosophila*	Whole flies	every 2–6 days throughout the life	DIMS	Carbohydrates, amino acids, carnitines, biogenic amines, lipids	[94]
	Whole flies	days 1–80	LC–MS	Lifetime dynamics of many metabolites	[51]
	Whole flies	days 3, 10, 24, 36, 51, 66, 81	LC–MS	Metabolism of carbohydrates, glycerophospholipids, neurotransmitters, amino acids, and the carnitine shuttle	[50]
	Heads, thoraces, abdomens, whole flies	days 10, 25, and 40	LC–MS	Metabolism of amino acids and NAD+	[106]
	Whole flies	days 4, 10, 24, 45, 69, 80	LC–MS	Arginine-ornithine metabolism, tryptophan metabolism	[104]
	Whole flies	day 3, day 30	LC–MS	Glycolysis	[107]
	Heads, muscle tissue	day 3, day 30	LC–MS	Carbohydrate metabolism (galactose, starch, sucrose metabolism), amino acids metabolism (alanine, asparagine, glutamine, serine metabolism), purine metabolism	[105]
Fishes	Blood plasma	2.4 ± 0.5 ^1^ years 3.4 ± 0.5 ^1^ years 6.7 ± 2.4 ^1^ years 4.3 ± 1.9 ^1^ years 6.1 ± 1.9 ^1^ years 4.0 ± 0.4 ^1^ years (from groups of short-lived to long-lived fish species)	DIMS	Dipeptides, di- and triglycerides, fatty acids, phosphoethanolamines, and phosphatidylcholines	[102]
	Skeletal muscles			Amino acids, lipids, biogenic amines, intermediates of glycolysis, glycogenolysis, and the citric acid cycle	[103]
Mice	Blood plasma, muscle tissue (quadriceps), liver	13 weeks (“young”), 93 weeks (“old”)	GC–MS, LC–MS	Metabolism of fatty acids and glucose	[62]
	Serum	8–129 weeks	LC–MS	Phospholipids, fatty acids, organic acids, creatine, methionine, uric acid	[108]
	Serum, urine	8, 12, 16, and 20 weeks (mutants with accelerated aging)	NMR spectroscopy	Changes in lipid and energy metabolism, transition to ketosis	[82]
Rats	Liver, serum	3–5 months (young), 15–17 months (old)	LC–MS	Organic acids and their derivatives, lipids and lipid-like molecules, glycerophospholipids, arachidonic acid, histidine, linoleate	[109]
Dogs	Urine	13, 18, 32 weeks, 1, 1.5, and 2 years, annually after 5 years until the death	NMR spectroscopy	Metabolites associated with energy metabolism	[98]
	Serum	1 month-16 years	NMR spectroscopy	Lipids, cholesterols, triglycerides, lipoproteins, protein glycosylation marker GlycA	[110]
Humans	Blood plasma	20–65 years	LC–MS, GC–MS	Tricarboxylic acid intermediates, creatine, essential and non-essential amino acids, urea, ornithine, polyamines, markers of oxidative stress, lipid metabolism products (including fatty acids, carnitine, β-hydroxybutyrate, and cholesterol), dehydroepiandrosterone sulfate (putative antiaging androgen), xenobiotics (e.g., caffeine)	[111]
	Whole blood, blood plasma, and erythrocytes	29 ± 4 ^1^ years (young), 81 ± 7 ^1^ years (elder)	LC–MS	1,5-anhydroglucitol, dimethylguanosine, acetylcarnosine, carnosine, ophthalmic acid, UDP-acetylglucosamine, N-acetylarginine, N6-acetyllysine, pantothenate, citrulline, leucine, isoleucine, NAD+, and NADP+	[95]
	Blood plasma	6 months–82 years	LC–MS	Metabolism of progestin steroids, xanthine, and long-chain fatty acids	[58]
	Blood plasma	every two years from middle-aged adults for 10 years	LC–MS	Sphingolipids, lipid steroids (including androgens, progestins, and pregnenolones), amino acids	[112]
	Blood plasma, serum	17–85 years	LC–MS	Lipids (long-chain fatty acids, polyunsaturated fatty acids, and other fatty acids), amino acids (including glutamine, tyrosine, histidine)	[113]
	Serum	60.51 ± 8.77 ^1^ for females, 61.17 ± 8.79 ^1^ for males	LC–MS, GC–MS	Amino acids, lipids (fatty acids, androgenic steroids)	[114]

^1^ mean ±s.d.; DIMS, direct infusion mass spectrometry; LC–MS, liquid chromatography–mass spectrometry; GC–MS, gas chromatography–mass spectrometry; NMR, nuclear magnetic resonance.

## Data Availability

Not applicable.
